# Dispersive silicon–nitride optical phased array incorporating arrayed waveguide delay lines for passive line beam scanning

**DOI:** 10.1038/s41598-022-23456-7

**Published:** 2022-11-05

**Authors:** Bishal Bhandari, Chenxi Wang, Ji-Yeong Gwon, Jin-Moo Heo, Sung-Yong Ko, Min-Cheol Oh, Sang-Shin Lee

**Affiliations:** 1 Department of Electronic Engineering, Kwangwoon University, Seoul, 01897 South Korea; 2 i3system, 60-3 Jang-Dong, Yuseong-Gu, Daejeon, 305-343 South Korea; 3 Department of Electronic Engineering, Pusan National University, 2 Busandaehak-Ro, 63beon-gil, Geumjeong-Gu, Busan, 46241 South Korea

**Keywords:** Photonic devices, Integrated optics

## Abstract

As optical phased arrays (OPAs), used as solid-state beam scanning elements, swiftly stride towards higher efficiency and faster scanning speed, the line beam scanner is emerging as a viable substitute for its counterpart relying on point-beam-incorporated raster scanning. However, line-beam scanners require active phase shifters for beam scanning; thus, they consume more power and have complex device designs. This study proposes and demonstrates a dispersive silicon–nitride OPA that allows for passive wavelength-tuned steering of a line beam with an elongated vertical beamwidth. To steer the line beam passively covering the two-dimensional field of view, we deployed an array of delay lines with progressive delay lengths across adjacent channels. Furthermore, adiabatic tapers that allow precise effective array aperture adjustment are used as emitter elements to flexibly realize different vertical beamwidths. Combinations of different delay-length differences and taper tip-widths resulted in beam coverage (lateral × vertical) ranging from 6.3° × 19° to 23.8° × 40° by tuning the wavelength from 1530 to 1600 nm. The main lobe emission throughput was as small as − 2.8 dB. To the best of our knowledge, the embodied OPA is the first demonstration of a passive line beam scanner facilitating an adjustable beam coverage with quick operation and enhanced efficiency.

## Introduction

The rapidly increasing demand for compact light detection and ranging (LiDAR) systems has triggered a surge in the research and development of solid-state beam-scanning mechanisms for various applications including remote sensing, surveying, and optical communications^1–4^. Although most beam scanners are pivoted on either microelectromechanical systems (MEMS) or optical phased array (OPA) technologies, the latter is more widespread owing to its complementary metal-oxide semiconductor (CMOS) compatibility, leading to a compact footprint^1,5^. OPAs exploit power splitters, phase shifters, and emitters to emit and steer the beam. The emitter configuration governs the nature of the beam emission, such as end-fire emission^6,7^ or out-of-plane emission^8,9^. Current OPA developments are towards out-of-plane emission for the two-dimensional (2D) raster scanning of a point beam covering the field-of-view (FOV) of concern by tuning the laser wavelength and phase distribution across emitter channels^1,3,8,9^. Typically, OPAs incorporate diffractive-grating-based emitters to enable longitudinal beam scanning by tuning the wavelength of the input light. Phase tuning across emitter channels using either the electro- or thermo-optic effect results in lateral beam steering. Although point-beam raster-scanning OPAs are widely adopted, they inevitably suffer higher excess losses owing to their grating-based emitters. Furthermore, these OPAs require active components, such as phase modulators with heaters or electrodes, and require electronic driver circuitry for their control, increasing the complexity of their fabrication and operation. The raster-scanning approach is slow, considering that the beam must be scanned through each pixel^3^.

As an alternative solution, line-beam-emitting OPAs, which do not involve diffraction grating-based emitters, resulting in higher emission efficiency, are slowly gaining attention^6,7,10^. However, these devices require active components for phase tuning to facilitate the beam scanning. Previous studies realized dispersive OPAs which could scan a point beam in both the longitudinal and lateral directions passively without involving any active phase modulators^2,11–13^. However, these OPAs still deployed grating emitters and raster-scanned the beam for each pixel in the FOV of interest, rendering slow and less efficient beam scanning. There are a few theoretical studies demonstrating dispersive OPAs without using grating emitters, but they incorporated multilayered waveguides, making them challenging to realize practically^14,15^. Hence, a line-beam scanning OPA that can realize passive beam scanning by exclusively using laser wavelength can overcome the above limitations.

In this study, a dispersive silicon–nitride OPA performing wavelength-tuned line beam scanning is demonstrated. It is fully passive and incorporates arrayed waveguide delay lines (AWDLs) for wavelength-tuned line beam scanning, whose length can be varied to tailor the beam scanning rate and range. The OPA is designed for the end-fire emission of a line beam with a larger beamwidth along the vertical direction. The line-beam, when scanned along the lateral direction can cover the entire two-dimensional FOV, eliminating the raster scanning constraint of each pixel in point-beam scanning OPAs, thereby quickening beam scanning. Furthermore, passive OPA achieves enhanced emission efficiencies by excluding diffraction grating-based emitters and active phase modulators^6^. Arrays of tapered waveguides have been used to implement a line beam emitter, with the intention of adjusting the beamwidth along the vertical direction. Because line beam scanning with AWDLs increases propagation loss due to the increased waveguide length, we adopted silicon nitride as the material of choice because of its low propagation loss and high-power handling capacity^16–19^. To the best of our knowledge, there has been no report on a dispersive OPA that enables passive line-beam scanning.

## Design of the proposed line beam scanning OPA

**Figure 1:**
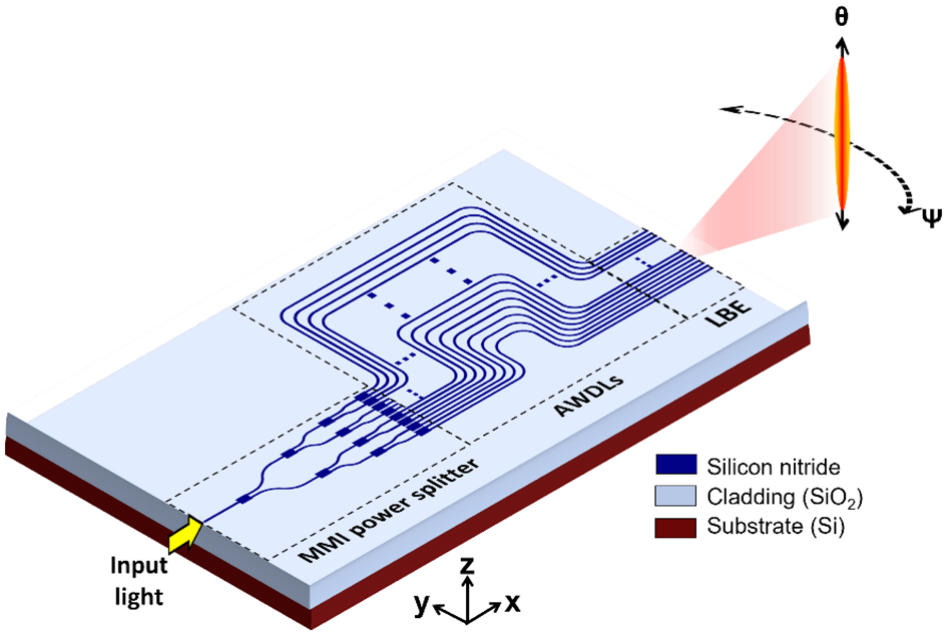
Illustration of the proposed integrated OPA incorporating the AWDLs involving an array of delay lines for wavelength tuned line beam scanning.

An illustration of the proposed OPA chip is shown in Fig. 1. It consists of an input spot size converter (SSC) that couples light from a laser source to the chip. The SSC is adjoined to a power splitter that uniformly distributes the input power into 32 output channels. The power splitter comprises 1 × 2 multi-mode interferometers (MMIs), which are serially cascaded in five stages to provide 32 output channels. The structural parameters pertaining to the MMIs and S-bend structures were determined using rigorous simulations, thereby achieving uniform power splitting. Each of the output channels is subsequently concatenated to AWDLs, which are tethered to a line beam emitter (LBE). The designs of the LBE and AWDLs are thoroughly discussed in the following sections. The waveguides are based on a 500-nm-thick SiN platform, which is placed on top of a silicon wafer loaded with a BOX layer with a thickness of 4 µm. The waveguides have a 3.3-µm-thick layer of SiO_2_ on top, acting as the upper cladding.

### LBE design

**Figure 2:**
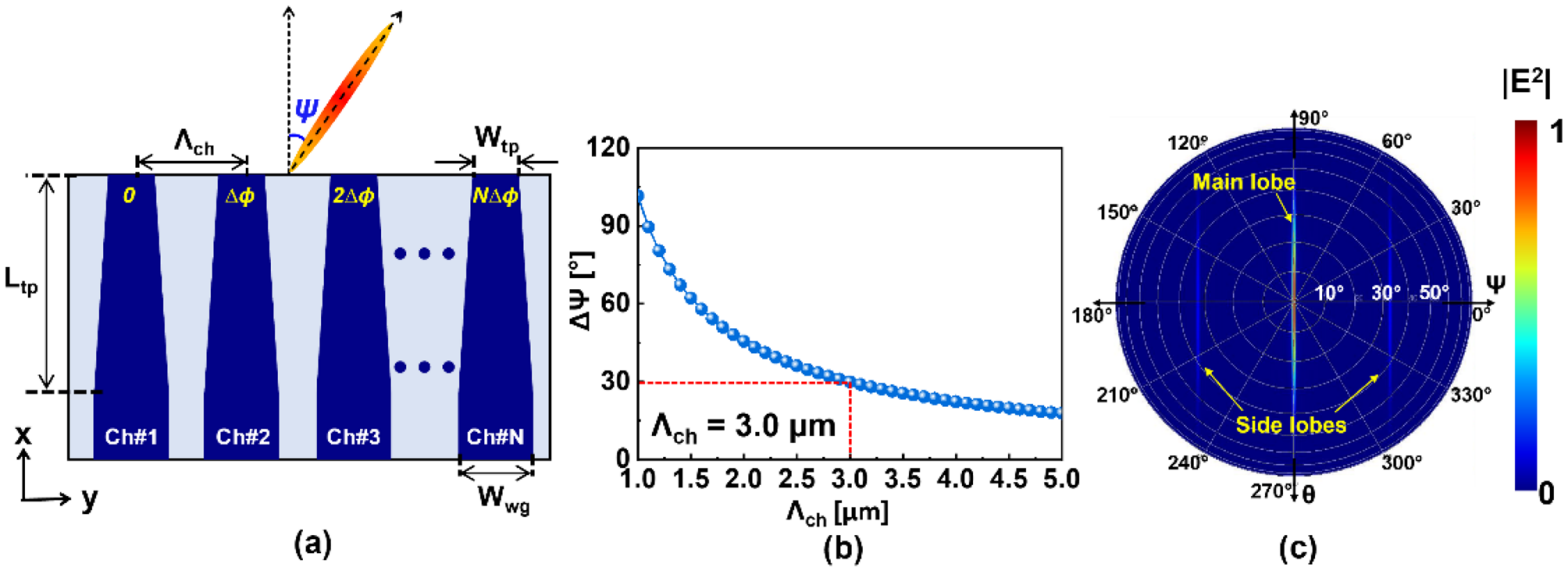
(**a**) Top view of the LBE indicating the emitter channels and the beam scanning parameters. (**b**) Calculated beam scanning range (ΔΨ) for the proposed end-fire OPA when $\Delta \varphi$ is varied between + π and − π. (**c**) The simulated far-field profile of the emitted line beam showing the main and side lobes emanating from a 32-channel OPA with ${\Lambda }_{{{\text{ch}}}}$ = 3 µm and W_tp_ = 500 nm.

For the proposed device, the LBE comprises an array of tapered waveguides as radiating elements, as shown in Fig. 2a. To achieve single-mode operation, SiN waveguides were designed by resorting to a width (W_wg_) and thickness of 1 µm and 500 nm, respectively^16,20^. The tapers acting as the LBE elements adjust the effective aperture at the end facet, facilitating a flexible full width at half maximum beamwidth (Δθ_FWHM_) along the vertical direction. The tapers corresponding to each emitter channel have a tip width (W_tp_) of 500 nm. A taper length (L_tp_) of 100 µm is sufficient to instigate lossless mode evolution. The beam is scanned by inducing a phase difference ($\Delta \varphi$) between adjacent channels to tune the lateral emission angle (Ψ).

The spacing between the emitter channels (${\Lambda }_{{{\text{ch}}}}$) and number of channels (N) are crucial parameters that determine the beam scanning range (ΔΨ), the position of the secondary side lobes, and the beamwidth along the lateral direction^1,3,13^. ΔΨ, which is the FOV along the lateral direction depend on ${\Lambda }_{{{\text{ch}}}}$. The maximum available scanning range in terms of ${\Lambda }_{{{\text{ch}}}}$ can be theoretically estimated using $\Delta \Psi = 2\sin^{ - 1} (\lambda / 2\Lambda_{{{\text{ch}}}} ),$ for $\Delta \varphi$ varying from − π to + π, as plotted in Fig. 2b^11^. The scanning range increases with decreasing ${\Lambda }_{{{\text{ch}}}}$, which might incur channel crosstalk to exacerbate the beam characteristics^5,21,22^. In contrast, increasing ${\Lambda }_{{{\text{ch}}}}$ could avoid crosstalk but result in a reduced ΔΨ, which is undesirable for extending the FOV. Considering the trade-off between ${\Lambda }_{{{\text{ch}}}}$ and ΔΨ, a ${\Lambda }_{{{\text{ch}}}}$ of 3 µm was adopted, leading to an FOV of ± 15° along the lateral direction. Finally, to achieve a lateral beamwidth (ΔΨ_FWHM_) below 1°, 32 channels were chosen^11^. A 32-channel OPA was then simulated using a finite-difference time-domain (FDTD) tool (Ansys Lumerical Inc.). The refractive indices of the SiN and SiO_2_ used for the simulations were 1.98 and 1.44 at λ = 1550 nm, respectively. The far-field profile of the radiated beam is shown in Fig. 2(c), which indicates that a line beam has been formed with a wide vertical beamwidth of $\Delta \theta_{{{\text{FWHM}}}} = \sim 35^\circ$ and a narrow lateral beamwidth of $\Delta \Psi_{{\text{FWHM }}} = \sim 0.8^\circ$. The side lobes were observed to be positioned at $\Psi = \pm 31.1^\circ$, guaranteeing a lateral FOV of ± 15°, free from sidelobes during beam scanning.

### AWDL design

Phase modulators based on the thermo-/electro-optic effect are indispensable for an end-fired OPA for beam scanning along the lateral direction^6,10^. The beam can be scanned along the lateral direction according to $\sin \Psi = \lambda \varphi / (2\pi \Lambda_{{{\text{ch}}}} )$. However, the deployment of an active modulator requires an additional circuit to drive current or heat into it, leading to phase control, which makes the OPA more complex and energy intensive. Alternatively, we attempted to exploit a passive scheme of AWDLs to induce $\Delta \varphi$ between emitter channels. The phase difference can be introduced by delaying the light through a length ΔL between consecutive channels in accordance with $\Delta \varphi = 2\pi \Delta Ln_{{{\text{eff}}}} /\lambda$, where n_eff_ is the effective refractive index of the guided mode of the waveguide for the delay line at a wavelength of λ^23^. Consequently, $\Delta \varphi$ is dependent on λ and n_eff_, rendering the beam scanning initiated through the waveguide dispersion characteristics, realizing passive dispersive line beam scanning.

**Figure 3:**
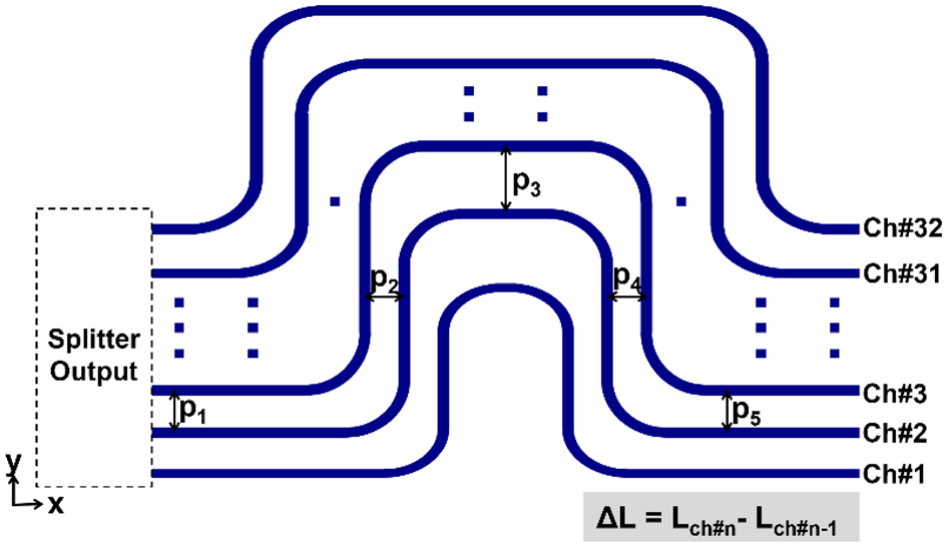
Configuration of the proposed AWDL structure enabling dispersive line beam scanning, with all the structural parameters indicated. Here, ΔL is the difference in length between adjacent channels.

AWDLs should be precisely determined from the perspective of the channel length difference ΔL for the beam scanning characteristics. A schematic of the AWDL structure is shown in Fig. 3. Each delay line mimics a horseshoe-like (Ω) structure incorporating a series of right-angle bends and straight waveguide segments, the length of which is progressively altered across the channels to obtain a uniform discrepancy of ΔL between adjacent channels. A group of right-angled bends with a radius of 100 µm was used to attain a lossless optical connection. The spacings between the adjacent channels at different sections of the delay lines are labeled p_1_, p_2_, p_3_, p_4_, and p_5_, from the power splitter interface toward the end of the delay line. The values of p_1_ and p_5_ were fixed at 3 µm, corresponding to the spacing associated with the power splitter and emitter. To alleviate design complexity, the values of p_2_ and p_4_ were maintained at 3 µm. Hence, ΔL is exclusively dependent on p_3_. The design of the AWDL is such that $\Delta L = L_{{{\text{ch}}\# n}} - L_{{{\text{ch}}\# n - 1}}$, for any value of channel ‘n’. For an OPA governed by AWDLs with a length difference ΔL, the beam emission angle can be estimated using^11,13,24^

$$\sin \Psi = {\text{m}}\frac{\lambda }{{\Lambda_{{{\text{ch}}}} }} + \frac{{{\text{n}}_{{{\text{eff}}}} \Delta {\text{L}}}}{{\Lambda_{{{\text{ch}}}} }} ,$$

where, $m = - q$ is the integer closest to $q = \Delta L n_{{{\text{eff}}}} /\lambda$, representing the diffraction order of the configuration of the arrayed emitters. The designed AWDLs closely resemble the delay lines in arrayed waveguide gratings, whose order is ‘q’^25^. Assuming angle Ψ to be small, the beam scanning rate (${\text{d}}\Psi /{\text{d}}\lambda$) pertaining to the AWDLs is approximately governed by the following relation:

$$\frac{{{\text{d}}\Psi }}{{{\text{d}}\lambda }} \approx \frac{{{\text{d}} \sin \Psi }}{{{\text{d}}\lambda }} = \frac{{\text{m}}}{{\Lambda_{{{\text{ch}}}} }} + \frac{{{\text{dn}}_{{{\text{eff}}}} }}{{{\text{d}}\lambda }}\frac{{\Delta {\text{L}}}}{{\Lambda_{{{\text{ch}}}} }}$$

The negative value of m is indicative of beam scanning towards the negative Ψ direction with increase in wavelength. Consequently, a larger value of m results in a longer delay length difference ΔL. To explore the beam-scanning characteristics, an OPA employing AWDLs was designed and analyzed using theoretical calculations and FDTD simulations. The calculated waveguide dispersion in the form of ${\text{d}}n_{{{\text{eff}}}} /{\text{d}}\lambda$ for a SiN waveguide with a thickness of 500 nm and a width of 1 µm was ~ 2.5 × 10^–4^/nm. For the OPA based on AWDLs with $\Delta L = 13$ μm the theoretical value of ${\text{d}}\Psi /{\text{d}}\lambda$ was − 0.33°/nm. The results obtained from the FDTD simulations agreed with the calculated results, as anticipated. Because the delay lines induced a phase gradient rather than a fixed phase distribution across the channels, the OPA performance has been mainly assessed by referring to the beam scanning rate.

### Experimental characterization of line beam scanning OPA

To evaluate the proposed line-scanning OPA, a suite of samples was fabricated on a 100-mm silicon wafer coated with a 4-µm-thick SiO_2_ layer. A 500-nm-thick SiN layer was subsequently deposited using low-pressure chemical vapor deposition (LPCVD), which was then etched to carve out the waveguide patterns. A 3.3-µm-thick film of SiO_2_ was formed via plasma-enhanced chemical vapor deposition (PECVD) on top of the waveguides to function as the upper cladding. The created samples were dry-etched to dice out the chips and expose edge facets, thus facilitating excellent fiber-to-waveguide coupling. Several OPAs with different delay lengths were fabricated in conjunction with straight waveguides, bent waveguides, SSCs, and MMI splitter structures. These structures were utilized to measure the propagation loss, SSC loss, and the loss per MMI splitter, which were observed to be 0.24 dB/cm, 1 dB/SSC and 0.13 dB/MMI, respectively. The right-angled bends were tested to incur a loss of 0.02 dB/bend, which is negligibly small and hardly hampers the operation of the OPA.

**Figure 4:**
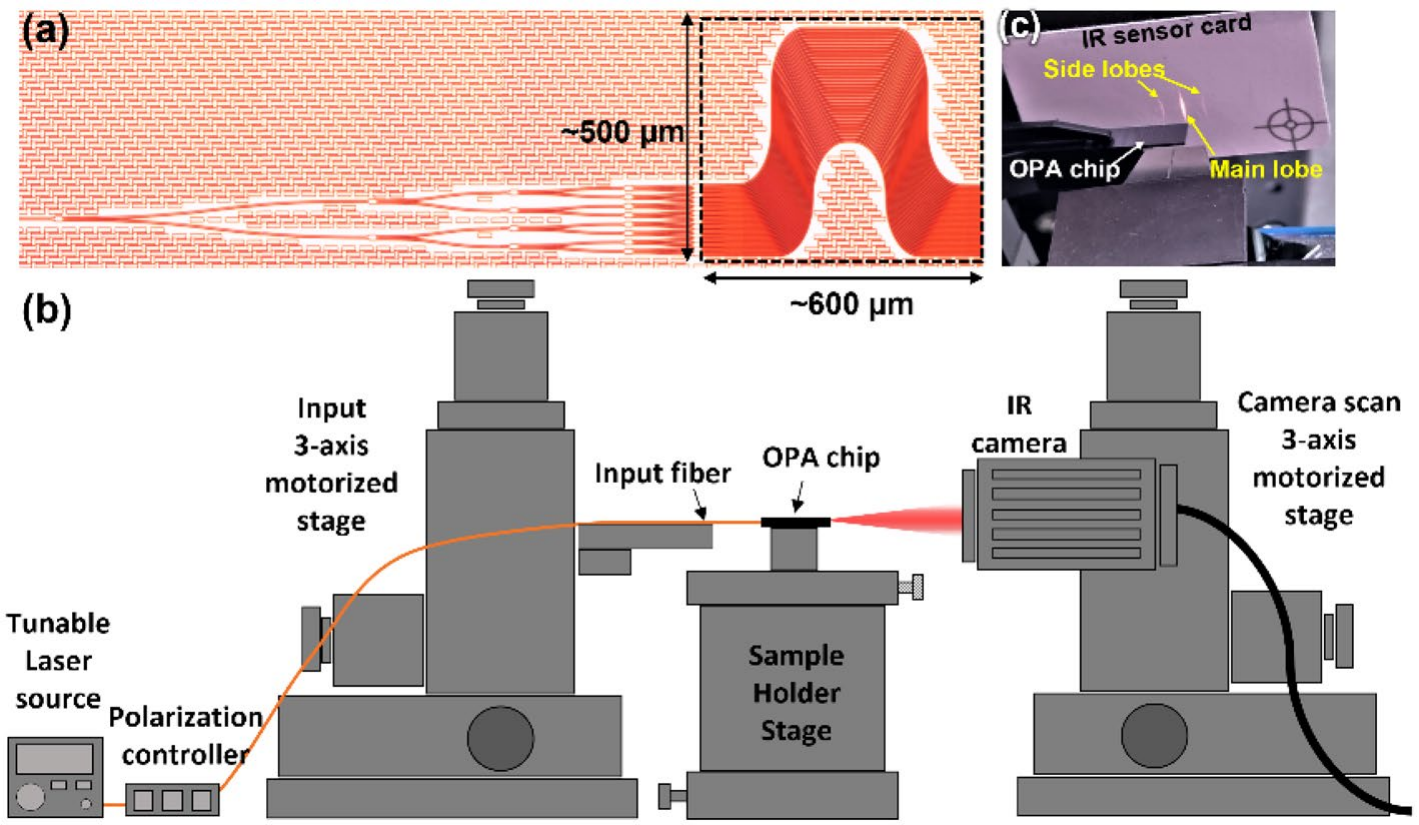
(**a**) A microscopic image of the fabricated OPA incorporating an AWDL. (**b**) The experimental setup used to characterize the OPA, and (**c**) the beam emitted from the fabricated OPA as seen in an IR sensor card.

**Figure 5:**
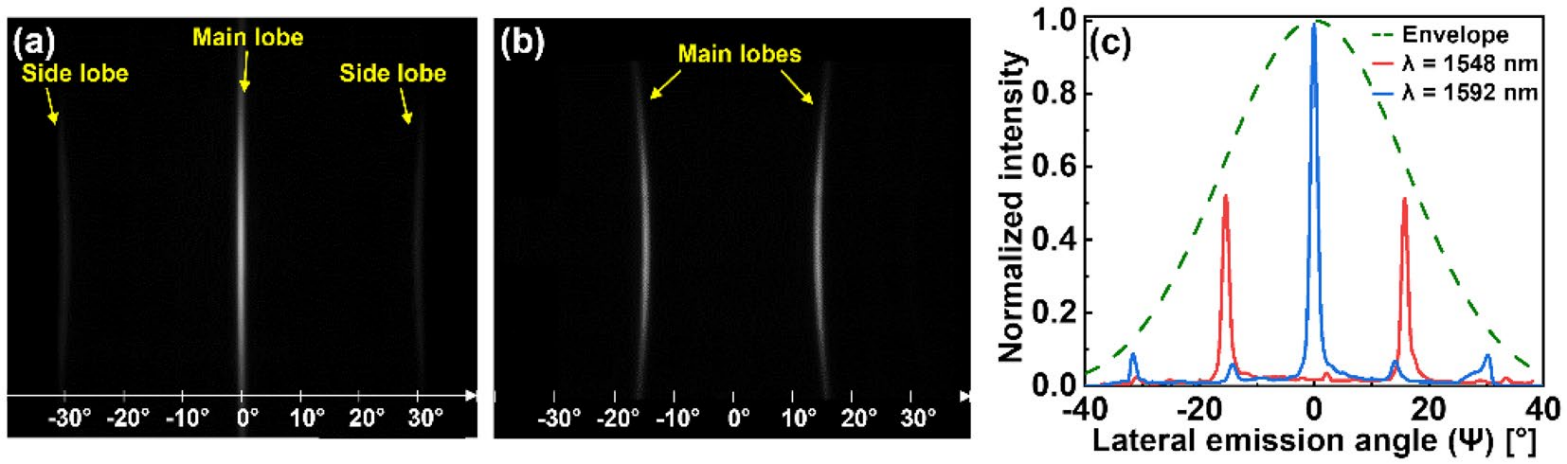
Images of the emitted line beam from the manufactured OPA when (**a**) λ = 1592 nm, where $\Delta \varphi$ was found to be 0, and (**b**) λ = 1548 nm, where $\Delta \varphi$ was found to be π. (**c**) The cross-sections of the images shown in (**a**) and (**b**) along with a cross-section of the beam emitted from a single emitter element, i.e., a taper with W_tp_ = 500 nm. The measured envelope is fitted using Gaussian function approximation.

A microscopic image of the prepared OPA is shown in Fig. 4a. The OPA was evaluated using the experimental setup shown in Fig. 4b. Light emanating from a tunable laser source (TLS) [WSL-110, Santec] was transited through a polarization controller to set the transverse-electric polarization. Light was then fed into the chip via the SSC. The emerging beam was first observed using an infrared (IR) sensor card, as shown in Fig. 4c. As expected, the corresponding main lobe resembled a line beam and the first-order side lobes were concurrently visible. To record the beam emanating from the chip, a short-wave IR (SWIR) camera [ABA-001IR-GE, AVAL Data] was placed on a 3-axis motorized stage to scan the area of the overall beam with a large magnitude of Δθ_FWHM_. Considering that the absolute phase distribution relating to the AWDLs constituting the fabricated OPA is practically unknown, the angle of emission could not be readily predicted using simulations. Experimentally, for the OPA with a set of AWDLs with $\Delta L = 13$ μm, the main lobe was observed to be located at Ψ = 0° at a wavelength of 1592 nm, corresponding to the case of $\Delta \varphi = 0$, as shown in Fig. 5a. The measured Δθ_FWHM_ and ΔΨ_FWHM_ values of the beam were ~ 34° and ~ 0.8°, respectively. Some residual scattered beams with substantially lower intensities than the side lobes were observed in between the main and the side lobes, which are attributed to the fabrication induced phase errors. As shown in Fig. 5b, the OPA yielded a main lobe at $\Psi = \sim \pm 15^\circ$ at a wavelength of 1548 nm, translating into the case of $\Delta \varphi = \pm \pi$. Figure 5c shows the lateral cross-section of the beams, as observed at the two wavelengths, alongside the envelope of a single emitter element, which is tantamount to a simple taper with W_tp_ = 500 nm. Overall, the lateral cross-sections corresponding to the beams for both Ψ = 0° and $\pm 15$° agree with the envelope, signifying appropriate beamforming and scanning.

**Figure 6:**
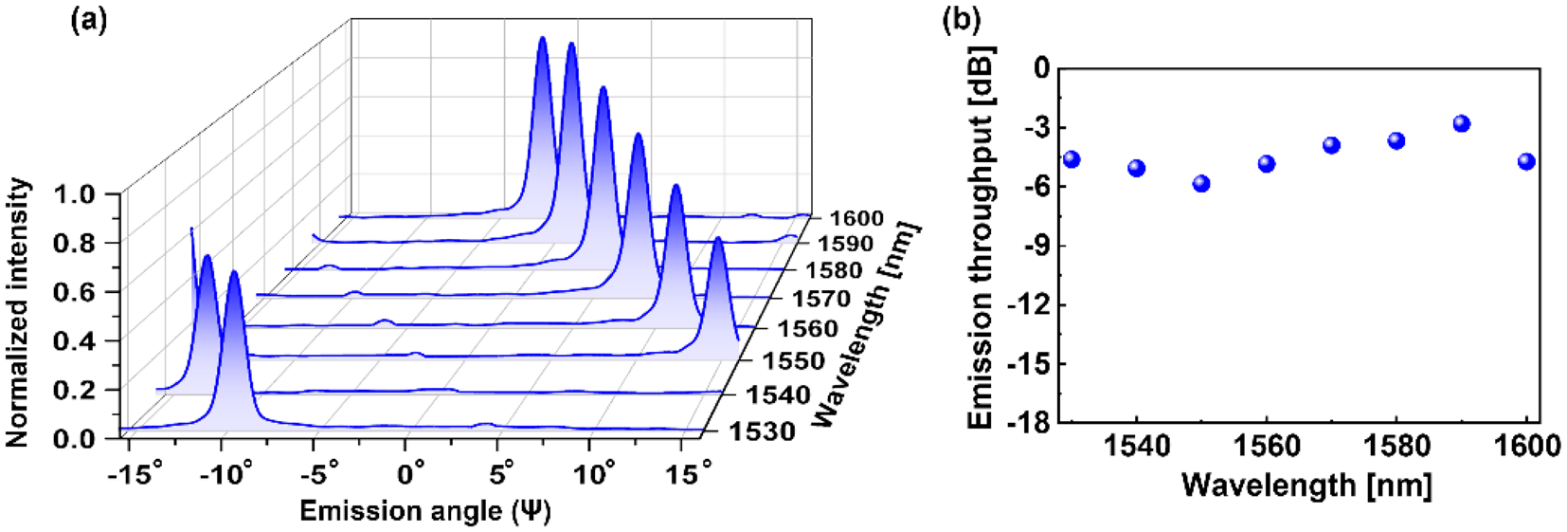
(**a**) Lateral cross-sections of the line beam at different wavelengths ranging from λ = 1530 nm to 1600 nm, covering the C and L bands. The measured ${\text{d}}\Psi /{\text{d}}\lambda$ was 0.34°/nm, resulting in a total scanning range (ΔΨ) of ~ 23.8°. (**b**) The measured throughput of the emitted main lobe. The throughput is maximum at a wavelength where the beam is emitted in the vicinity of Ψ = 0° at λ = 1590 nm and is minimum for the case of Ψ =  ~ 15° at λ = 1550 nm.

The beam scanning characteristics of the OPA capitalizing on AWDLs with $\Delta L = 13$ μm was then inspected in terms of line beam scanning characteristics by changing the wavelength, as shown in Fig. 6a. Considering the stepwise wavelength tuning of the TLS instead of continuous tuning, the spectral emission characteristics have been measured for a set of discrete wavelengths covering the C and L spectral bands with an interval of 10 nm. The main beam was found to be steered towards the negative Ψ direction with an increase in the wavelength until it reached the delay line-induced $\Delta \varphi$ of ± π at λ = 1548 nm. At this wavelength, two identical beams were observed at $\Psi = \pm 15^{^\circ }$. With a further increase in the wavelength, the beam at $\Psi = - 15^{^\circ }$ shifted towards the negative ${\Psi }$ direction away from the ${\Lambda }_{{{\text{ch}}}}$ constrained lateral FOV, with a diminishing intensity according to the envelope. In contrast, the beam at $\Psi = + 15^{^\circ }$ steered towards the negative ${\Psi }$ direction within the lateral FOV limits with increasing intensity for increased wavelengths, in accordance with envelope.

Hence, the steered beam from $\Psi = + 15^{^\circ }$ becomes the main beam for longer wavelengths, rendering a high-intensity main beam always within the ${\Lambda }_{{{\text{ch}}}}$ limited FOV. For the OPA driven by AWDLs with $\Delta L = 13$ μm, the beam was found to steer at a rate of − 0.34°/nm, resulting in a total scanning range of ~ 23.8° for wavelengths covering the C and L bands (λ = 1530–1600 nm). The measured steering range is commensurate with the calculated rate of − 0.33°/nm. The main-lobe emission throughput, which is the total coupling efficiency from the fiber to the main lobe of the emitted beam, was found to be − 2.8 dB at λ = 1590 nm for $\Psi ={\text{}} \sim 0^\circ$, as shown in Fig. 6b. With beam scanning, the main lobe power dropped and reached − 5.8 dB at *λ* = 1550 nm approximately corresponding to $\Psi = \pm 15^{^\circ }$, which is in good agreement with the expected power variation of − 2.4 dB based on the envelope intensity. The discrepancy between the expected intensity variation and the measured power variation could be attributed to the elongated nature of the beam with a wide vertical beamwidth, hindering the exact power measurement. Nevertheless, the emitted power is superior to the main lobe power of grating-based OPAs involving similar SiN waveguides^3,8,20^. It is worth mentioning that the intensity drop during beam scanning depends on the envelope width, which can be widened by increasing the W_tp_ or incorporating plateau envelope-forming emitters involving an oxide offset between the emitter and chip edge^7^. It is worth mentioning that the power per unit area (intensity) of the emitted line beam might be lower as compared to the grating-based counterpart^3^. However from the perspective of deploying a vertical array of photodiode pixels for detection of the incoming beam, the decreased optical intensity can be acceptable as long as each photodiode pixel responds to a segment of incoming beam impinging upon the pixel. Further, the decreased optical intensity can be compensated for to some extent by effectively enhancing the throughput of the proposed line-beam-based OPA incorporating no-grating structure.

### Construction of an OPA serving flexible beam coverage

**Figure 7:**
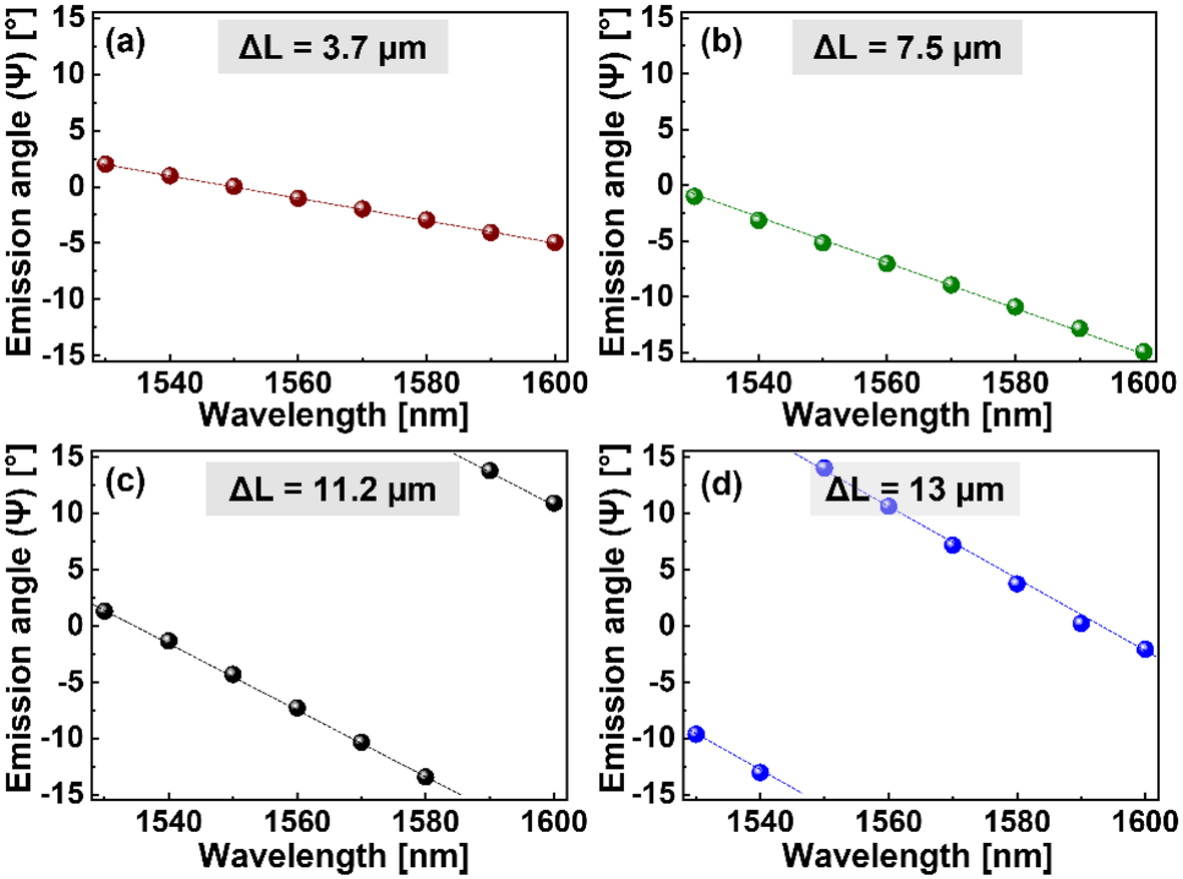
The measured emission angles of OPAs incorporating different AWDLs corresponding to (**a**) $\Delta L = 3.7$ μm, (**b**)$\Delta L = 7.5$ μm, (**c**) $\Delta L = 11.2$ μm and (**d**) $\Delta L = 13$ μm.

Considering that their delay lengths can directly affect the beam scanning characteristics, AWDLs could be meticulously designed to tailor the FOV for a fixed wavelength range, ultimately varying the beam coverage. To investigate the effect of AWDLs on the beam scanning rate, several OPAs with different AWDL configurations were fabricated and characterized. The measured emission angles of the OPA corresponding to AWDLs with $\Delta L = 3.7$ μm, 7.5 µm, 11.2 µm, and 13 µm are shown in Fig. 7a–d, respectively.

The value of ${\text{d}}\Psi /{\text{d}}\lambda$ was observed to increase with ΔL, exhibiting rates of 0.09°/nm and 0.34°/nm for AWDLs corresponding to $\Delta L = 3.7$ μm and $\Delta L = 13$ μm, respectively. The beam scanning is continuous towards negative ${\Psi }$ for the length difference resulting from $\Delta L = 3.7$ μm and $\Delta L = 7.5$ μm; however, the emission angle abruptly appears in the region of positive ${\Psi }$ when the beam exceeds the FOV limit of $\Psi = - 15^{^\circ }$ for $\Delta L = 11.2$ μm and $\Delta L = 13$ μm. As explained in the experimental characterization section, for a beam exceeding the negative FOV limit, the beam at positive ${\Psi }$ becomes dominant in intensity and acts as the main beam, resulting in an abrupt change in the emission angle. It is worth mentioning that the scanning rate is insensitive to the dimensions of the taper and the number of channels. Hence, implementing different tip widths or taper lengths could result in a different emission angle owing to the change in absolute phase values; however, because the relative $\Delta \varphi$ for beam scanning is exclusively governed by ΔL, the scanning rate remains unchanged.

All the measured and calculated beam scanning characteristics corresponding to the OPAs with AWDLs of different delay lengths are tabulated in Table 1. The free spectral range (FSR), which is a measure of the wavelength required to achieve Δφ from − π to + π, decreases with ΔL. As the FSR and ΔΨ_FWHM_ values determine the scanning resolution along the lateral direction^2,23^, they should be appropriately chosen according to the application requirements. It should be noted that for the dispersive OPA incorporating AWDLs for achieving passive beam steering, the FSR should be equal to or smaller than the wavelength sweep range to cover the maximum possible FOV^13^. In light of the targeted lateral FOV over 20°, an AWDL with ΔL = 13 µm, leading to a FSR of 88.1 nm, was chosen to achieve a lateral FOV of ~ 23.8°. Since the maximum FOV is limited by the emitter ${\Lambda }_{{{\text{ch}}}}$, the increased scanning rate will result in repetitive scanning within the FOV limits. Although the maximum scanning range is constrained by ${\Lambda }_{{{\text{ch}}}}$, the OPA can be readily engineered for different lateral FOVs under a fixed wavelength range by adjusting the delay lengths of the AWDLs.

**Table 1:** Beam scanning rates in terms of the ΔL.

ΔL [µm]	m	Beam scanning rate (dΨ/dλ)		FSR [nm]
		Calculated [°/nm]	Measured [°/nm]	
3.7	− 4	0.09	0.09	332.6
7.5	− 8	0.19	0.19	157.6
11.2	− 12	0.28	0.29	103.2
13	− 14	0.33	0.34	88.1

**Figure 8:**
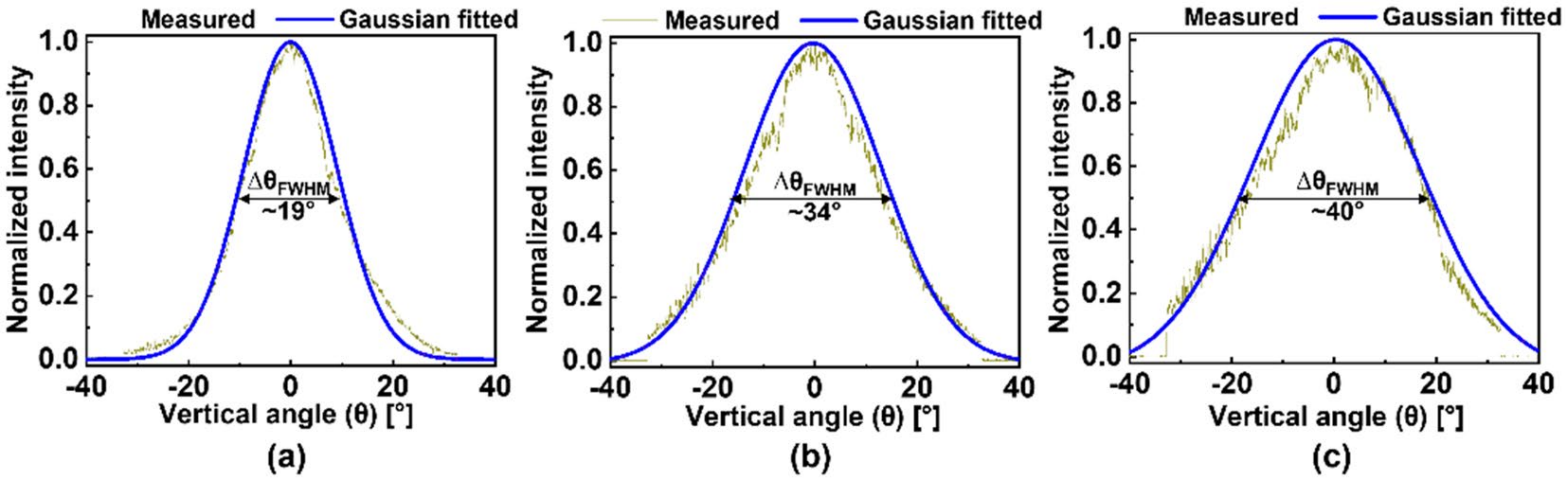
Measured vertical cross-sections of the radiated line beam at Ψ = 0°, for an OPA with tapers of (**a**) W_tp_ = 300 nm, (**b**) W_tp_ = 500 nm, and (**c**) W_tp_ = 1000 nm. The measured raw data are fitted with reference to a Gaussian function. The vertical beamwidths are indicated by ∆θ_FWHM_.

The proposed OPA incorporates an array of tapered emitters, which facilitates the adjustment of the vertical beamwidth, thus altering the vertical beam coverage. Because the far-field beam width depends on the effective near-field beam profile^11^, the vertical beam coverage can be tailored by controlling the vertical near-field beam profile. For an OPA catering to line-beam scanning, the vertical near-field profile is chiefly determined by the waveguide thickness. However, inverse tapers are known to significantly expand the beam in both the vertical and lateral directions^20^, thereby changing the vertical beamwidths. Because the aperture size in the lateral direction is the cumulative sum of the beamwidths of multiple channels, the beamwidth change becomes negligible in comparison to the wide array width. Hence, the beamwidth adjustment based on tapered emitters is more pronounced in the vertical direction than that in the lateral direction. As a proof of concept, a few OPAs with different ${\text{W}}_{{{\text{tp}}}}$ were additionally prepared. The measured vertical beam cross-sections of the OPAs in response to ${\text{W}}_{{{\text{tp}}}}$ = 300 nm, 500 nm, and 1000 nm at $\Psi = 0^{^\circ }$ are shown in Fig. 8a–c, respectively. The measured $\Delta {\uptheta }_{{{\text{FWHM}}}}$ were as small as ~ 19° for the OPA with ${\text{W}}_{{{\text{tp}}}} {\text{ = 300 nm}}$ and as high as ~ 40° for ${\text{W}}_{{{\text{tp}}}} {\text{ = 1000 nm}}$.

Finally, the OPAs with various ΔL and tip widths were assessed to examine the possible beam coverage area. The vertical beamwidth of the emitted beam indicates the vertical coverage area, and the lateral beam coverage area is equivalent to the lateral FOV, which is the effective scanning range under a fixed spectral range of λ = 1530−1600 nm. The measured beam coverage area for the different OPA adopting different combinations of AWDL delay lengths and W_tp_ are listed in Table 2. An OPA for the combination of a smaller $\Delta L = 3.7$ μm and a smaller width of W_tp_ = 300 nm gave rise to a beam coverage area having 6.3° × 19° (lateral FOV and vertical beamwidth). The beam coverage area could be enlarged to 23.8° × 40° for an OPA incorporating AWDL with a longer $\Delta L = 13$ μm in conjunction with a W_tp_ of 1000 nm. Based on these results, the proposed OPA could be readily devised to achieve adjustable beam coverage area along the lateral and vertical directions by appropriately tailoring AWDLs and W_tp_, respectively. Hence, the proposed OPA can potentially be deployed towards the development of passive photonic beam scanners with enhancements in terms of reduced power consumption, flexible coverage area, and high speed.

**Table 2:** Beam coverage area (lateral FOV × vertical beamwidth) in terms of W_tp_ and ΔL.

ΔL ⇒	3.7 μm	7.5 μm	11.2 μm	13 μm
W_tp_ ⇓				
300 nm	6.3° × 19°	13.3° × 19°	20.3° × 19°	23.8° × 19°
500 nm	6.3° × 34°	13.3° × 34°	20.3° × 34°	23.8° × 34°
1000 nm	6.3° × 40°	13.3° × 40°	20.3° × 40°	23.8° × 40°

## Conclusion

In summary, a dispersive OPA incorporating arrayed delay lines was developed to achieve highly efficient and passive line-beam scanning. All components, including the LBE and AWDLs, were meticulously designed to emit and steer the line beam. By incorporating tapered emitters, the vertical beam width of the line beam can be adjusted, enabling a flexible vertical FOV. Because the emitters did not possess diffractive elements as in conventional OPAs, the proposed OPA could attain an excellent main lobe throughput of − 2.8 dB. By appropriately selecting the combination of the taper tip width and ΔL, the proposed OPA can be flexibly designed to scan a wide range of coverage area. In the future, to further increase the beam coverage area, longer delay lines with smaller ${\Lambda }_{{{\text{ch}}}}$ can be implemented on a waveguide with reduced thickness. It is noted that scaling up the AWDLs may require a larger chip footprint leading to possibly deteriorated phase errors. To mitigate this issue, different types of delay-inducing structures may be practically applicable including snake delay lines or imbalanced splitter-based ones^2^. With its fast beam scanning, flexible FOV optimization, and higher emission efficiency, the proposed OPA is anticipated to pave new avenues towards the development of fully passive OPAs for high-power and faster beam scanning applications.

## Methods

A finite-difference eigenmode (FDE) solver [Ansys Inc.] were used to identify the effective refractive indices and field profiles for the theoretical calculation. The FDE solver solves the Maxwell’s equation across the meshed cross-sections of the waveguides and determines the mode field profiles and their effective refractive indices. To determine the length of tapers, a three-dimensional eigenmode expansion (EME) solver from Ansys Inc. was adopted. All other simulations were made using three-dimensional full-vectorial FDTD method. The refractive indices of the SiN and SiO_2_ were assumed to be 1.98 and 1.44 at a wavelength around 1550 nm, respectively for all the simulations.

## Data Availability

The datasets generated and/or analyzed during the current study are available from the corresponding author upon reasonable request.
